# Social media for health promotion and weight management: a critical debate

**DOI:** 10.1186/s12889-018-5837-3

**Published:** 2018-07-28

**Authors:** Monica Jane, Martin Hagger, Jonathan Foster, Suleen Ho, Sebely Pal

**Affiliations:** 1 Curtin University School of Public Health, Perth, Australia; 2 Curtin University School of Psychology and Speech Pathology, Perth, Australia; 3 Health Department of WA, Neurosciences Unit, Perth, Australia; 4 School of Public Health, Faculty of Health Sciences, Curtin University, GPO Box U1987, Perth, Western Australia 6845 Australia

**Keywords:** Health promotion, Obesity, Overweight, Social media, Social support, Weight management

## Abstract

**Background:**

In 2016 an estimated 1.9 billion adults world-wide were either overweight or obese. The health consequences of obesity are responsible for 2.8 million preventable deaths per year. The WHO now considers obesity as a global epidemic and recommends population-wide health promotion strategies to address this issue. Weight gain is caused by increased energy intake and physical inactivity, so treatment should focus on changes to behaviour regarding diet and physical activity.

**Discussion:**

The WHO has also recognised the importance of social resources as a valuable agent for behaviour change in health promotion. Social resources are translated at the community level as support provided by significant others such as family, partners and peers, in the form of information, material aid and encouragement. Social support has been shown to improve health and well-being, whereas social isolation has been shown to have a negative impact on health outcomes. Social support provided by peers has been shown to be a useful strategy to employ in weight management programmes. The documented increased use of ICT and social media has presented health promoters with a potentially useful medium to increase social support for weight management.

**Conclusion:**

While the use of social media for health promotion is an emerging field of investigation, preliminary research suggests that it increases participant engagement, and may provide a cost-effective tool to provide social support for individuals participating in weight management programmes. With stringent privacy protocols in place, social media may be a useful, cost-effective accompaniment to multifactorial weight management programmes. However more research is needed to identify how to make the best use of social media as health promotion tool.

## Background

According to the World Health Organisation (WHO) world-wide obesity has nearly tripled since 1975, and with over 1.9 billion overweight and obese adults worldwide identified in 2016 [1], obesity is now a global epidemic [2]. Should the upward trajectory of obesity continue unabated it may come to be considered by individuals as a new social norm. As a measure of public health, overweight is defined as a body mass index (BMI; measured as kg/m^2^) of 25 or more, and the BMI for obesity it is 30 or more [3]. Overweight and obesity are strongly related to behavioural risk factors, such as low levels of physical activity and intake of high energy diets. Obesity is associated with increased risk of cardio- and cerebro-vascular diseases, type 2 diabetes and some cancers [1, 3, 4]. The WHO estimates that globally 2.8 million people die every year as a consequence of obesity [5], a preventable condition [1], and as such recommend population-wide interventions as one of several strategies to address this problem [5].

Population-level interventions to prevent and manage obesity focus on changing the behaviours that are associated with increased risk of obesity, i.e. encourage healthy eating and regular physical activity. Such interventions rely on a number of strategies or techniques aimed at changing behaviour [6]. Utilising social support has been identified as a key technique that may assist in promoting health behaviour change. Social support can be described as: “the social resources that persons perceive to be available or that are actually provided to them by nonprofessionals in the context of both formal support groups and informal helping relationships” [7]. According to the Ottawa Charter for Health Promotion, in addition to access to health information and services, individuals also need the social resources to support healthful life practices [8]. Health promotion initiatives can facilitate health behaviour change by promoting support for individuals and groups from salient agents within their environment [9], such as family members, peers and friends. With the nature of interpersonal interactions evolving in line with advancing digital technology, interactive online platforms such as social media may prove to be an effective method of engaging social resources for health promotion interventions. The aim of this debate is to discuss the efficacy of health promotion interventions delivered via social media, particularly in the area of weight management.

## Main text

### Social support

Social support encompasses the provision of material resources, useful information, emotional care, and affirmative feedback, which promote health maintenance attitudes and behaviours [10]. Research has indicated that social support confers physical [11–13] and mental [14, 15] health benefits. Even the *perception* of support – the belief that help is available if needed - has been shown to have beneficial effects [7, 16]. In addition, a study of self-rated health and happiness conducted in Italy revealed a strong positive association between health and happiness, with the author suggesting that one reason for this is that happy individuals may be more inclined to engage in health-promoting behaviours [17].

Interpersonal communications are highlighted in research into social support and health [14, 16, 18], and are sometimes considered as mediators of behaviour change, although rarely evaluated within health promotion interventions [19, 20]. Interpersonal interactions can facilitate the delivery of potentially useful information or provide encouragement. ‘Shared experiences’ and like-mindedness can be empowering and promote self-efficacy [18, 21]. According to social learning theories, role modelling of healthy behaviours in social contexts can exert a positive influence on individuals’ behaviour [18, 21, 22] and improve health-promoting self-efficacy [21]. As far as weight management specifically is concerned, social influences and normative beliefs have been associated with weight status and intentions to lose weight among young adults with overweight or obesity [23]. Therefore, health promotion interventions that manage to positively influence social norms may have better and more sustainable outcomes [24].

In terms of weight management, research has shown that individuals who have social support are more likely to have better weight loss outcomes than those who do not [25]. Social groups have been shown to influence food behaviours in individuals [26] so engaging social support for dietary changes may have beneficial effects. The social support may include support from a family member, significant other, weight loss partner, or health professional. However many individuals attempting weight loss do not always receive the required social support for a variety of reasons [27]. Weight management studies that employed group-based interventions in either treatment versus standard care [28], or group versus individual treatment [29], have reported clinically meaningful weight loss from participants in all group conditions irrespective of intervention, at least in the short term [30, 31]. This suggests that belonging to a group may be just as useful as the actual treatment programme itself. An example of this is a study into social support within weight loss programmes compared participants recruited with one or more friends (also wishing to lose weight) to a control group following the same programme individually, and reported greater weight loss and weight loss maintenance for up to ten months in the ‘friends’ group [32]. In this study those participating with friends also showed better programme retention than the control group [32]. On the other hand, social network members can also provide negative role models. The long-term Framingham Heart Study - conducted between 1971 to 2003 - evaluated social interconnection within a cohort of 12, 067 individuals and found obesity to spread within ones’ social ties [33].

### Internet communication technologies

Advances in Internet Communication Technologies (ICT) have created new avenues for the delivery of health promotion interventions, especially as global internet coverage continues to increase. Presently there are well over 3.2 billion internet users world-wide, and counting [34]. In the fifteen years to November 2015, every world region – Africa, Europe, Asia, the Americas, and Oceania (includes Australia) – experienced growth in internet usage [35]. The mobile and (internet-enabled) Smart phone market had reached 96.2% globally, as at the first quarter of 2013, with Africa recording the lowest penetration at 63% [36], however the emerging nations represent a growth area for both mobile and internet services [37]. The implication of the increasing internet and mobile technology sector, as far as health promotion is concerned, is that information can now be accessed at home or away 24-h a day seven days a week, at the convenience of the individual.

As internet-provided information has the potential to reach large populations, and may also be able to access harder to reach groups [15], health promotion interventions have begun to incorporate this technology [38]. Although initially health promoters were reluctant to use ICT due to the fact that disadvantaged groups had limited access to it [39], the emerging view is that health promotion will benefit from support from online resources [40]. The interactivity and potential cost-effectiveness are additional benefits of the internet as a platform for health promotion. At the moment, internet access is a limiting factor for this approach as coverage is yet to reach 100%.

### Social media

Increased accessibility to ICT and advances in software and hardware for social interaction has given rise to a variety of social networking sites. Initially, *social networking sites* were solely internet-mediated communication services that allowed individuals to create public profiles with a list others in their online community and to have online interactions with those individuals [41]. Over time, the capabilities of these services have expanded, enabling users to send messages and share information with the online community. Most social networking sites are free to join, and some sites have gained widespread usage. The growth of Facebook membership is a good example of this. Between the first quarter of 2013 and the first quarter of 2015, steady growth in Facebook usage was recorded in Europe, Asia-Pacific, USA and Canada [42]. Overall, there are currently over 1.5 billion active Facebook users world-wide, and counting [43]. As with the internet, social networking sites can also take advantage of mobile technology, and could provide another avenue for health promotion interventions. The term *social media* will be used in this review as this term encompasses these additional capabilities of online social networking services.

While some health promotion interventions have incorporated an internet element [38], using social media, more specifically, may be better able to target population subgroups (including at risk or minority groups), because it offers direct access to existing online social networks [44]. A recent systematic review of health information presented on social media revealed that social networking sites were accessed by groups that are often hard to reach via traditional health promotion messages, such groups as those of low socioeconomic status (SES), young people and ethnic minorities [45]. Furthermore, research into the motivations for belonging to online support groups found ‘information seeking’ to be a primary motivation [46].

On the other hand, viewing content on social networking sites can negatively influence mood, via social comparison. For example, upward comparisons of content posted by individuals with higher financial, vocational or education attainment - perceived to be higher than the viewer - may damaging to mood and self-perception [47]. Frequency of social media use can also influence mental health; frequent usage has been linked to internet addiction which has been found to be positively associated with depression [48].

### Social media and health promotion

Studies have identified social media as a source of social support [49]. For example, a recent study revealed that individuals that reported experiencing social connectedness through Facebook had lower anxiety and depression, and greater subjective wellbeing [50]. Another study found that Facebook assisted individuals to *gain* support, which was significantly associated with offline social support and similarly associated with wellbeing [51]. It has also been suggested that online social networks may be able to influence social norms [44, 52], an important element of health promotion interventions [20].

A systematic review identified numerous studies reporting evidence that health promotion messages delivered via social media generated social support for patients and/or peers [45]. The desire to connect with others in similar circumstances was another strong motivator for belonging to an online support group [46]. One study of 299 Facebook users found that socially anxious individuals were better able to derive social support online than offline [53]. In addition, helping others has been identified as another primary motivation for belonging to online support groups [46]. Helping each other has been found to be mutually beneficial, as the helper also benefits by feeling or becoming more useful or less dependent [21].

Interactive, online health promotion campaigns for specific health issues have been shown to provide social support for behaviour change. A study of health issue-specific social media sites for smoking cessation has shown that active participation – regularly sharing information and experiences, asking/answering questions - exerted a significant positive influence on smoking cessation self-efficacy, improved social capital, perception of similar or shared social norms and of feeling supported socially [54]. However as participation was by self-selection, the positive results may have been achieved by the more active or ‘successful’ participants within the group. The results of a pre-test, post-test examination that compared an online smoking cessation campaign to a conventional ‘Smoker’s Helpline’ recently conducted in Canada showed that the online intervention had double the smoking cessation rates to that of the helpline; further, the availability of at least one support person was predictive of successful smoking cessation in both groups although this was not significant statistically [55].

Targeted, online health promotion campaigns have also demonstrated the capacity to reach large numbers of participants. The above-mentioned study attracted 44, 172 hits to the site, by 37, 325 individual visitors within the three-month campaign period [55]. It seems that these results are not limited to social networking for smoking cessation. A study that used Facebook to deliver sexual health promotion messages attracted approximately 900 fans (or ‘likes’) and a total 5309 views of the 31 promotional videos posted during a five-month period [56]. A US intervention to address declining condom use in young adults that used Facebook for recruitment and to deliver health promotion messages to participants, found Facebook to be effective in promoting condom use in the short term (2 months), and suggest that it may be a useful avenue for recruitment (*n* = 1578), however both effect and retention declined over the longer term [57].

In summary, it appears that individuals not only seek health information on the internet, but also have specific health-related motives for joining interactive online support groups. As these studies show, social media is effective at providing a support to network members. They also indicate that health promotion campaigns delivered via social media have the potential to attract large numbers of participants and demonstrate a certain level of engagement with the message.

### Social media and weight management

Prior to the emergence of ICT the self-help approach – following a diet or programme without professional help or support - offered the lowest cost weight management strategy [21]. It has been speculated that web-based weight management programmes may provide another cost-effective alternative to conventional therapies (e.g. in-person weight management treatments). One study examined the differences in cost per person between two group weight loss programmes, one delivered in-person and the other via group internet ‘chat’ sessions, and while both groups demonstrated clinically meaningful weight loss, the in-person group showed significantly better weight loss than the internet group by the end of the six month intervention period [58]. On the other hand, economic analysis revealed that not only did the internet-delivered weight management programme cost less per person it also cost less per kilogram lost [58].

As weight regain after weight loss is very common [4], the cost of continuing care needs to be taken into account after weight loss. A weight loss maintenance trial was conducted following successful weight loss, compared the cost-effectiveness of a ‘personal contact’ programme to an internet-delivered programme, and found the internet-delivered programme to cost less, however only the in-person treatment produced statistically significant weight loss [59]. Social media may be an even less expensive avenue, particularly if an existing platform (e.g. Facebook) is used for programme delivery. The interactivity of social media together with user-generated content may provide a friendly environment and enhance the outcomes of such interventions.

#### Online interactivity

Including an interactive component to an intervention may be especially helpful for intractable public health conditions such as obesity, and may provide cost-effective and accessible weight management interventions [60]. A systematic review and meta-analysis investigated behavioural physical activity (PA) and dietary weight management interventions that utilised social networking features and noted that message board services provided as a part of internet-delivered interventions were the most commonly used [61]. The analysis failed to find statistically significant differences in primary outcomes (such as weight, BMI and PA levels), and in the few studies that reported improvements, the changes were not maintained [61]. However, these findings may be the result of inconsistencies between the intervention protocols, as there was a fair degree of heterogeneity between the trials reviewed. Furthermore, the studies where reductions to BMI or weight were primary outcomes, this review reported a variety of weight management programmes, of varying durations, across different age ranges, and was not limited to participants with a high BMI only [61].

A systematic review and meta-analysis of weight management trials that incorporated elements of online social networking found a small, but statistically significant reduction in BMI in the intervention groups at six months, which tapered off by 12 months, which is similar to findings in conventional weight loss interventions [60], where weight regain after initial weight loss is a common phenomenon [4]. Furthermore, there were no significant differences in any of the other primary measures (such as blood lipid profiles, body fat composition, weight, waist circumference and blood pressure), and participant retention was found to be problematic [60]. Again, the degree of heterogeneity between study protocols may have influenced these results. Of note, social support was not reported in neither this study nor the study conducted by Williams and colleagues (noted above), so it is unclear if participants were encouraged to make use of social networking features in this way.

While systematic reviews provide a broad overview of the specific interventions types under investigation, the finer details of the studies reviewed often escape analysis. An assessment of individual studies that have used social media to assist with weight management shows that many interventions have used a combination of ICT as a part of the study protocol, including Twitter, Facebook, text messaging, podcasts, and mobile phone apps. In addition, these studies have sometimes included contact with a trained professional via such media, usually text messages. A weight loss trial using Twitter for social support with programme content delivered via podcasts and mobile phone app, found that degree of usage was positively associated with weight loss although it was not clear whether Twitter use assisted weight loss or participants losing weight ‘shared’ more, as not all participants engaged with Twitter, and usage declined over time [62]. Interestingly, participants’ Twitter posts were examined for elements of social support with the results showing that informational support was predominant, emotional support increased over time, however there was no evidence of material support [62]. Similarly, a study of the Weight Watchers Facebook page to examined members’ perceptions of social support reported that the majority of members derived both informational and emotional support through the site [63], however weight loss data was not collected in this study.

On the other hand, some social media weight management trials reported a statistically significant intervention effect i.e. weight loss, but did not report on the degree or type of social support received by participants. One such study used Facebook to provide weight management programme content (including podcasts), together with text messaging to programme strategy reminders, recorded weight loss in the intervention group [64]. Participants were also required to have a support ‘buddy’ from outside of the study group, and received personalised feedback, but even so social support was not reported [64]. Another similar trial used text messaging for two-way communication between a trained health coach and participants - to send weight management strategies and receive participants’ progress reports – with Facebook for social support, reported weight loss in the intervention group [65]. Despite having a Facebook group and personalised feedback from the health coach social support was not measured in this study [65]. Contrary to the majority of weight management interventions, participant retention was high in both this and the previously-mentioned study, however both trials were of relatively short duration [64, 65].

Judging by the studies reviewed, weight management interventions that utilise social media tend to measure either changes to weight and other risk factors or level of social support, but rarely the two together. By including a combination of ICT within the same intervention the exact cause/s of the resultant outcomes can be difficult to determine. In order to more fully understand how to make best use of social media as a vehicle for weight management interventions, emerging research has begun to examine changes to weight and other risk factors alongside outcome measures associated with social support, using one social media platform only e.g. Facebook [66].

Due to the complex nature of weight loss and regain [4], social support provided via social media may be a useful tool to include in multifactorial weight management interventions. Research cited in this review demonstrates that social media may be an acceptable supplement or even substitute for offline social support for individuals undergoing health behaviour changes (including weight management), and may be important especially if offline social support is inadequate. Similarly, information sharing can be gained by belonging to social media support groups, and this may provide a useful supplementary service for participants between visits to their health care practitioners, especially where distance can make accessing services difficult. Participant retention seems to be problematic, but this issue is not limited to weight management [21] or social media [67] interventions. Until the root cause/s of participant attrition are identified, high drop-out rates will continue to plague weight management interventions, regardless of how they are delivered.

#### Potential pitfalls

The ability of users to generate and/or share content may create the potential to disseminate incorrect information [45, 60], which could apply to any intervention conducted without professional involvement [21]. Social media can also be a distraction, leading to the overconsumption of food [68]. In addition, food posts have been shown to make an individual’s desire eat when not hungry [68]. Considering the ubiquity and accessibility of ICT, protecting the privacy, data and confidentiality of intervention group members is another concern that has been raised [49]. Some researchers have speculated that engaging with unknown individuals may be a relevant concern [44], while other researchers have suggested that the generation/use of health-specific sites may allay some of these concerns, as participants may feel they can trust those that they can relate to [54]. Others have suggested that participants’ online privacy should be treated with same confidentiality as when patients receive professional health care treatments in conventional settings [45]. Some sites such as Facebook have addressed this issue with group privacy settings that can be set to ‘secret’, so all group content is only visible to group members [69]. Therefore, instead of generating and administering dedicated social networking sites and attempting to attract new members to it, a more practical and financially prudent approach may be to utilise existing social media platforms and user networks, with appropriate caveats and constraints, as the target group may already be within reach [70]. Educating health professionals, patients and the public about social media may help to address issues surrounding privacy concerns as well as those arising from disseminating incorrect information [45].

## Conclusions

The world-wide prevalence of obesity bears testament to the intractability of the issue. That traditional health promotion interventions have failed to have an impact thus far is sufficient evidence for the urgent need for improved weight management interventions. Social support is important for the maintenance of good health, and is equally important when individuals undergo health-related behaviour changes, including weight loss. The use of social media for health promotion and weight management is still very much in the formative stages. Emerging evidence supports the use of social media to augment health promotion interventions, by providing social support to those undergoing health behaviour changes. It would seem that social media can provide participants with social support and/or assist with improving health outcomes (including weight loss), however studies to date have typically examined either health outcomes, or social support, but rarely the two together. In addition, many trials have used a combination of ICTs, making it the cause/s of the resultant outcomes unclear. More research is needed to determine whether incorporating social media into a weight management programme will assist individuals with overweight and obesity to achieve greater improvements in weight loss and other outcome measures than attempting dietary and lifestyle modifications on their own. Research is also needed to elucidate the particular aspects of social media that assist individuals with overweight and obesity to achieve improvements in weight and other outcome measures, to maximise the potential benefits of this relatively inexpensive tool.

## Abbreviations

BMI: Body mass index;
ICT: Internet communication technologies
PA: Physical activity
SES: Socioeconomic status
WHO: World Health Organisation
